# Detecting Suicidal Ideation on Forums: Proof-of-Concept Study

**DOI:** 10.2196/jmir.9840

**Published:** 2018-06-21

**Authors:** Ahmet Emre Aladağ, Serra Muderrisoglu, Naz Berfu Akbas, Oguzhan Zahmacioglu, Haluk O Bingol

**Affiliations:** ^1^ Department of Computer Engineering Bogazici University Istanbul Turkey; ^2^ Amazon Research Madrid Spain; ^3^ Department of Psychology Bogazici University Istanbul Turkey; ^4^ Medical School Department of Psychiatry Yeditepe University Istanbul Turkey; ^5^ Medical School Department of Child and Adolescent Psychiatry Yeditepe University Istanbul Turkey

**Keywords:** suicide, suicidal ideation, suicidality, detection, prevention, classification model, text mining, machine learning, artificial intelligence, suicidal surveillance

## Abstract

**Background:**

In 2016, 44,965 people in the United States died by suicide. It is common to see people with suicidal ideation seek help or leave suicide notes on social media before attempting suicide. Many prefer to express their feelings with longer passages on forums such as Reddit and blogs. Because these expressive posts follow regular language patterns, potential suicide attempts can be prevented by detecting suicidal posts as they are written.

**Objective:**

This study aims to build a classifier that differentiates suicidal and nonsuicidal forum posts via text mining methods applied on post titles and bodies.

**Methods:**

A total of 508,398 Reddit posts longer than 100 characters and posted between 2008 and 2016 on SuicideWatch, Depression, Anxiety, and ShowerThoughts subreddits were downloaded from the publicly available Reddit dataset. Of these, 10,785 posts were randomly selected and 785 were manually annotated as suicidal or nonsuicidal. Features were extracted using term frequency-inverse document frequency, linguistic inquiry and word count, and sentiment analysis on post titles and bodies. Logistic regression, random forest, and support vector machine (SVM) classification algorithms were applied on resulting corpus and prediction performance is evaluated.

**Results:**

The logistic regression and SVM classifiers correctly identified suicidality of posts with 80% to 92% accuracy and F1 score, respectively, depending on different data compositions closely followed by random forest, compared to baseline ZeroR algorithm achieving 50% accuracy and 66% F1 score.

**Conclusions:**

This study demonstrated that it is possible to detect people with suicidal ideation on online forums with high accuracy. The logistic regression classifier in this study can potentially be embedded on blogs and forums to make the decision to offer real-time online counseling in case a suicidal post is being written.

## Introduction

### Background

Suicide was the tenth leading cause of death for all ages in 2016 with 44,965 completed suicides in the United States, corresponding to 123 deaths by suicide per day [1]. According to estimations, approximately 2.7 million US adults (1.1% of the population) made a suicide plan in 2014. Among these, 1.3 million made an actual suicide attempt [2,3]. It was shown that suicide plans were more commonly (2.5%) observed among adults aged between 18 and 25 years [2]. Considering the high social media penetration rates of this group, many suicide attempts could possibly be prevented via social media surveillance. Suicide does not only affect the victim, but also their family, friends, and even society. The economic cost of death due to suicide in the United States in 2013 was estimated to be US $58.4 billion [4]. To prevent suicides, monitoring is significantly important. Risk factors of suicide include previous suicide attempt(s), history of depression or other mental illness, alcohol or drug abuse, family history of suicide or violence, physical illness, and feeling alone [2]. There is no agreement on the definition of the suicide stages yet [5]. However, suicidal people were commonly divided into two main classes: ideators (ideators, planners) and attempters (attempters, completers). Considering 80% of the patients attempting suicide were found to score in the depressed range [6], we consider depression a potential precursor to suicidal ideation: depression, suicidal ideation, plan, attempt, and completion.

### Suicidal Surveillance and Suicide Prevention

While monitoring for suicide prevention, it is prudent to “cast a wider net,” meaning it is fine to see a few false positive overhead, up to an acceptable level, for the sake of not missing suicidal people. It has been shown that half of all suicides are likely to occur in lower-risk groups [7]. In this sense, sensitivity/recall (the ability to accurately detect suicidal behavior) is more important than precision (the ability to accurately discard nonsuicidal behavior) and accuracy (predicting suicidal status correctly) [8]. However, having 100% recall and very low precision (which is unacceptable) would be a waste of resources and potentially annoying for the patient. Thus, a balance between the two should be sought.

Any suicidal sign should be taken seriously and the patient should be questioned on existence of suicidal thoughts. In common clinical practice, patients with depressive symptoms are asked whether they have any suicidal thoughts for early diagnosis. To perform a better risk assessment, suicide prevention researchers analyze patient history, statements, and suicide notes. It is known that more than 50% of suicide completers and 20% of suicide attempters left suicide notes [9]. Thus, leaving suicide notes should be considered a significant signal [10-13].

### Suicidal Ideation on Social Media

In the past, suicidal individuals could only write suicide notes to express their feelings and some studies focused on linguistic and content analysis of these notes [14]. However, with the introduction of social media, such as Facebook, Twitter, Reddit, and Tumblr, Internet users are now sharing their suicidal feelings and thoughts on these platforms [15,16]. Because postings occur in the earlier phases of ideation, these people can potentially be saved if proper support is given. For each post on such platforms, the following questions can be asked:

1. Does the author of this post have suicidal ideation?
2. Does this person have potential to attempt suicide?
3. Is this post a suicide note?
4. Is this post authentic?
5. Has this person already committed suicide?

Answering each question is a different problem on its own. Because every individual with suicidal or depressive expressions should be provided support, answering the first question (which is our objective) is more beneficial in suicide prevention. For this purpose, detecting suicidal and potentially suicidal people via surveillance is important.

### Text Mining Methods for Suicidal Ideation Detection

In efforts of suicidal surveillance, applying text processing and supervised machine learning (classification) techniques for performing suicidal text detection is becoming more popular in suicide research. In this approach, textual features are extracted from posts for discriminating suicidality of a text. Then statistical classification algorithms, such as logistic regression, random forest, and support vector machine (SVM) algorithms are applied to discover patterns (relationships between the features and the suicidality status). Finally, models resulting from training with these classifiers are evaluated with test data and evaluation metrics. Accuracy, precision, recall (sensitivity), and F_1_ score are the commonly used metrics for evaluation of the classifier performance. In suicidal post classification context, accuracy represents the fraction of posts classified correctly over all posts. Recall represents the fraction of suicidal posts that are correctly classified as suicidal. Precision represents the fraction of posts that are actually suicidal among the posts classified as suicidal. The F_1_ score is the harmonic mean of precision and recall, leading to a more balanced evaluation because precision and recall are complementary metrics.

The prediction performance heavily relies on extracting the best features. Several techniques are used for extracting significant features (feature extraction). Some are the bag-of-words model [17], term frequency-inverse document frequency (tf-idf) [18], linguistic inquiry and word count (LIWC) [19], and sentiment analysis. These techniques provide an analysis of words, themes, or tones commonly used in suicidal posts. See Multimedia Appendix 1 for a detailed explanation of these techniques.

### Related Work

There have been efforts on differentiating suicide note content from regular content [20-22]. With the rapid rise of social media, recent studies have begun to utilize text mining on online posts with depression [22], suicidal ideation [23-30], and mental health disorders [30]. These studies have shown the potential of using online posts to assess suicide risk or depression in English (Twitter) and Chinese (Weibo). However, character limitations make the prediction on these microblogging platforms error-prone because thoughts are spanned over multiple posts, making it harder to grasp the context if posts are evaluated independently during the machine learning process. On the other hand, evaluating all posts of a user results in a dilution of suicidality [25] because suicidality is not expected to be expressed in all posts. Therefore, there is a need to analyze all consecutive posts by the author and define boundaries of a suicidal set of posts. Moreover, recent studies on microblogging platforms used limited number of short posts and few of them provided sufficient performance. After further improvements, classifiers in the study by Guan et al [25] can be used for passive surveillance on Weibo to track users with suicidal mood spanning over a long time period, whereas classifiers in the study by O’Dea et al [24] can be used to detect impulsive suicidal expressions on Twitter. However, it was seen that strongly concerning suicide-related tweets and Weibo posts had higher word count [27,28]. This indicates that seriously suicidal individuals may need longer space to express themselves, such as blogs, forums, or Facebook posts. Thus, classifiers resulting from these studies may fall short in detecting serious and thoughtful suicidal ideators. Furthermore, longer posts have higher chance of being identified correctly due to their longer content. At this point, using Reddit as the dataset and aiming to predict suicidality on longer-form posts may be more effective.

### Goal of This Study

This study aims to build a classifier that detects long passages like forum and blog posts containing suicidal ideation via text mining methods to assist authorities in preventing potential suicide attempts.

## Methods

Classification models were developed to predict whether a given post with title and body text contains suicidal ideation using a dataset consisting of Reddit posts. The performance objective was to achieve a prediction performance that would mimic a human expert.

### Data Collection

In this study, a dataset containing publicly available Reddit posts was used. Reddit was chosen as the data source because it allows longer posts and has a special section on suicidal ideation. However, the generated models can be applied on blogs or any other social media platform, especially ones allowing long posts. No personally identifiable information apart from usernames (in many cases not revealing the real identity) and explicitly stated information were provided with the data. Nevertheless, usernames were not downloaded from the data source during this study and ethics committee approval was not sought. As a consequence, posts from the same authors were handled as separate posts. Because we did not have concerns about differentiating the author-post relationship, this limitation did not pose a problem.

Using Google Cloud BigQuery, posts with a text body of at least 100 characters and that were posted on the subreddits SuicideWatch, Depression, Anxiety, and ShowerThoughts between September 2008 and October 2016 inclusive (508,398 posts) were downloaded. Each post had an ID, title, body, and subreddit name. SuicideWatch is a subreddit where thousands of people write about their suicidal ideations. The majority of authors on this subreddit are depressed and thinking about suicide. They share their feelings and some ask for help. It is unknown whether any of these people killed themselves unless they left comments stating they changed their mind or published new posts afterwards. Nevertheless, the contents of these posts can definitely be seen as signs of suicidal ideation. Posts on the subreddits Depression and Anxiety contain depressive and anxious thoughts, respectively. A minority of posts on these two subreddits may contain suicidal thoughts as well because suicidal people may have anxiety and depressive feelings, which may lead them to write in these places. Posts on ShowerThoughts, on the other hand, contain authentic personal thoughts that came to mind in the shower on any topic. Therefore, ShowerThoughts is a good candidate for comparison against the aforementioned thought-oriented subreddits and not many suicidal posts are expected on this subreddit.

### Data Annotation

Among the posts collected, random posts were selected from all subreddits and manually annotated. In total, 785 posts on SuicideWatch (n=175), Depression (n=200), Anxiety (n=200), and ShowerThoughts (n=210) were manually annotated. Because the dataset would be balanced by binary annotation using oversampling, a balance in the number of annotated posts among subreddits was not sought. A post was labeled as suicidal (1) if the author of the post clearly seemed to have suicidal thoughts; otherwise, it was labeled nonsuicidal (0). One exception to this rule was posts on the SuicideWatch subreddit with strong depression and anticipated suicide risk. These posts were annotated as suicidal even if they did not have a suicidal language because posting on that subreddit is an implicit sign of suicidality. Posts on SuicideWatch were annotated by psychiatrists (NBA and OZ) with an initial agreement rate of 93% and a Cohen kappa [31] coefficient of κ=.74. The conflicts were then resolved by these authors reaching a consensus. It was seen that 150 of 175 (85.7%) posts were actually posts of people with suicidal ideation; 25 of 175 (14.3%) posts were not. The nonsuicidal segment contained (1) posts asking what to do for a suicidal friend, (2) posts of people who had a suicidal ideation in mind but who were not willing to die anymore, (3) posts asking questions about suicidal people, and (4) a few posts unrelated to the topic. These 25 posts seemed suicidal at first by solely looking at their choice of words; however, they were not suicidal although having been about suicidality. There were posts in a similar situation in other subreddits as well. Although these posts may cause noise when used as a test set, we included them in our dataset for better generalization. Posts in other subreddits were annotated by the AEA under consultancy and guidance of NBA and OZ. See Table 1 for distribution of the suicidality label among annotated posts.

**Table 1:** Suicidality label distribution of posts in subreddits.

Subreddit	Nonsuicidal, n	Suicidal, n	Total, n
SuicideWatch	25	150	175
Depression	152	48	200
Anxiety	193	7	200
ShowerThoughts	210	0	210
Total	580	205	785

### Dataset Formation

Four experiments were carried out with different samplings from four subreddits. For each experiment (*E*_i_), a custom dataset (*D*_i_) and a corresponding label vector (*L*_i_) indicating binary suicidality status was generated. The custom dataset contained post information from selected subreddits (or annotated post set) with rows corresponding to posts, columns corresponding to ID, subreddit, title, and body fields for posts. The label vector L_i_=[l_1_, l_2_,...,l_mi_]^T^ was a vector where *l*_j_ was the binary label for the corresponding post (*p*_j_). The label value was set l_j_=1 if the corresponding post *p*_j_ was annotated as suicidal, l_j_=0 otherwise (see Table 2).

### Feature Extraction

First, two features were extracted for all posts: LIWC matrices (*W*_t_ and *W*_b_) for title and body, and sentiment matrices (*S*_t_ and *S*_b_) for title and body. These were the constant features that did not change by composition of posts in datasets. Then, specifically for each dataset *D*_i_ to be used in *E*_i_, two more features were extracted: document term matrix for title (*T*_it_) and document term matrix for post body (*T*_ib_). See Multimedia Appendix 2 for a diagram of feature extraction and the experiment design steps.

#### Linguistic Inquiry and Word Count Matrix

Initially, LIWC 2015 tool [19] was run on all 508,398 posts (on titles and bodies separately), producing two LIWC matrices (W_t_ and W_b_) where rows corresponded to posts and columns (of size 93) corresponded to LIWC features. Each cell contained a calculated feature score for a post. Feature scaling (standard normalization) was applied on these scores to have all the features in the same range. Then, for each experiment *E*_i_, subsamples of the resulting matrix were extracted for each dataset to contain only rows that also existed in *D*_i_, resulting in *W*_it_ and *W*_ib_.

#### Sentiment Matrices

To build sentiment score matrices—*S*_it_ (for title) and *S*_ib_ (for body) for *D*_i_ dataset—Python TextBlob library [32] (which uses Python Natural Language Toolkit [NLTK] library [33] internally) was incorporated. This process yielded two augmented matrices S_it_=[S_itp_|S_itj_] and S_it_=[S_ibp_ |S_ibj_] each with two columns: polarity (*P*) and subjectivity (*J*) in the range [–1,1].

#### Document Term Matrices

To build *T*_it_ and *T*_ib_ matrices*,* title and body fields in *D*_i_ were used. For each row of *D*_i_, text in title/body field was converted to lowercase and applied the Porter stemming algorithm [34] with the NLTK library [33] to obtain the word stems. This allowed words to be evaluated in their canonical forms. Words of stem “suicide” were ignored in all subreddits to avoid classifying solely by existence of the word “suicide.” Then tf-idf document term matrices *T*_it_ and *T*_ib_ were built using Python scikit-learn library [35]. Having a large vocabulary (number of columns), one-way analysis of variance (ANOVA) F-test [36] was applied to the matrices to reduce the number of features to 200 for each of the two matrices, leaving the most important columns. This reduced the time required to train models with the classification algorithms.

#### Combining Features

At the end, these features were concatenated, resulting in corpus C_i_=[W_it_|W_ib_|S_it_|S_ib_|T_it_|T_ib_] with 590 columns: 93, 93, 2, 2, 200, and 200, in respective order, for each dataset *D*_i_. These corpora were combined with corresponding label vectors previously tied to *D*_i_, forming an augmented matrix [C_i_|L_i_] (see Table 3).

**Table 2:** Hypothetical dataset (D_i_) matrix and corresponding label vector (L_i_) for an experiment with two sample posts. A table in this form was generated for each experiment with different posts.

D_i_				L_i_
Post ID	Subreddit	Title	Body	Label
1	SuicideWatch	I don’t wanna live anymore	Since the day I was born,...	1
2	ShowerThoughts	Why are the oceans blue?	I have always wondered...	0

**Table 3:** Sample table representing concatenated C_i_ | L_i_ matrix containing 590 corpus feature columns (C_i_) plus one label column (L_i_) that were provided to machine learning algorithms for classification. A matrix in this form was generated for each experiment with different posts.

Post ID^a^	W_it1_…W_it93_^b^	W_ib1_…W_ib93_^c^	S_itp_^d^	S_itj_	S_ibp_^e^	S_ibj_	T_it1_…T_it200_^f^	T_ib1_…T_ib200_^g^	L_i_
1	0.3…0.00	0.15…0.22	-0.75	0.70	0.25	0.35	0.15…0.54	0.14…0.32	1
2	0.11…0.08	0.00…0.00	0.20	0.90	-0.45	0.78	0.07…0.93	0.01…0.63	0
Column #	1…93	94…186	187	188	189	190	191	391…590	1

^a^Post IDs are hypothetical.

^b^W_it_: Linguistic inquiry and word count (LIWC) matrice for title.

^c^W_ib_: LIWC matrix for body.

^d^S_it_: sentiment score matrix for title.

^e^S_ib_: sentiment score matrix for body.

^f^T_it_: document term matrix for title.

^g^T_ib_: document term matrix for body.

### Experiment Design

Each subreddit contained posts with different levels of suicidality. SuicideWatch mostly contained suicidal posts, Depression contained highly depressive and partly suicidal posts, and Anxiety contained some suicidal but mostly nonsuicidal posts. ShowerThoughts contained mostly nonsuicidal posts. Four experiments were conducted with different compositions of posts to see if discrimination for different levels of suicidality was possible. A new data table was generated for each experiment (see Table 4).

Experiment 1 was designed to see if it is possible to differentiate suicidal posts from posts talking about random daily matters. For this purpose, 175 annotated posts from SuicideWatch and 210 annotated posts from ShowerThoughts subreddits were selected because they are on different sides of the suicidality scale and provide good samples for contrast. To avoid a potential overfit, the experiment was evaluated with 10-fold cross-validation. This experiment was expected to yield good results because the two subreddits were expected to have mostly different vocabulary.

Experiment 2 was designed to see if it is possible to differentiate suicidality when posts with anxious/depressive vocabulary are involved. For this purpose, 200 Anxiety subreddit and 200 Depression subreddit posts, which can be seen as some of the closest psychological moods to suicidality, were included in addition to the composition of experiment 1, forming the second experiment. Because the vocabulary use of depressive, anxious, and suicidal people are expected to have commonalities and posts with these moods are harder to classify, a performance loss was expected in this experiment when compared to experiment 1. However, the diversity of the posts made the models in this experiment a finer-grain predictor in real-life applications.

Experiment 3 was designed to see if it is safe to assume all posts in SuicideWatch are suicidal (l_j_=1) and all posts in ShowerThoughts are nonsuicidal (l_j_=0) when training a model. For this purpose, models were trained with randomly selected, nonannotated 5000 SuicideWatch and 5000 ShowerThoughts posts under this assumption. The trained models were then tested against 175 SuicideWatch and 210 ShowerThought posts, which were already annotated to see if the model trained under the aforementioned assumption could perform well against the gold standard. Because the majority of SuicideWatch posts tend to be suicidal and the majority of ShowerThoughts posts tend to be nonsuicidal, only a slight performance loss was expected when compared to experiment 1.

Experiment 4 was designed to battle-test our model trained with the assumptions in experiment 3 against all 785 annotated posts including the depressive and anxious posts, which are difficult to make judgment on. Because the model was not trained with difficult cases, it was inevitable for it to fail in such cases. However, the models were still expected to perform better than the baseline model trained with ZeroR algorithm.

### Model Training and Evaluation

In experiments 1 and 2, rows (posts) from selected subreddits were appended, resulting in datasets *D*_1_ and *D*_2_. The datasets were then applied feature extraction steps to result in corpus *C*_1_ and *C*_2_. 10-fold cross-validation was applied on C_1_ and C_2_ and their corresponding label vectors *L*_1_ and *L*_2_. In each split, random synthetic minority oversampling technique (SMOTE) [37] was applied before training to obtain an equal number of posts from both classes and avoid imbalanced data bias. Models for each fold were trained with ZeroR (set to always classify posts as suicidal), logistic regression (delta=1.0) and random forest (with 10 trees) and SVM (with radial basis function kernel) classification implementations in Python scikit-learn library. To reduce fluctuations in scores (due to randomization and limited number of samples), experiments were repeated 100 times. Average metric scores were then evaluated. Logistic regression was chosen due to its efficiency, interpretable nature, ability to provide probabilities, and online learning (ability to update model parameters after being exposed to new labeled data) support. Random forest and SVM were chosen due to their high classification performance, especially for datasets with high number of instances and features. ZeroR was chosen as the baseline classifier for comparison.

**Table 4:** Summary of post distribution used in experiments (E).

Subreddit	Whole data (10-fold) posts, n			Train data posts, n		Test data posts, n	
	E1	E2	E3		E4	E3	E4
SuicideWatch	175^a^	175^a^	5000		5000	175^a^	175^a^
ShowerThoughts	210^a^	210^a^	5000		5000	210^a^	210^a^
Depression		200^a^					200^a^
Anxiety		200^a^					200^a^

^a^Annotated post.

In experiments 3 and 4, rows of train and test data were initially appended and followed the feature extraction steps. After building *C*_3_ and *C*_4_, rows were split (preserving the train/test formation) to form train and test corpuses *C*_train-i_ and *C*_test-i_ together with corresponding label vectors *L*_train-i_ and *L*_test-i_ for i=3,4. After training models on *C*_train-i_ and *L*_train-i_ with the aforementioned algorithms, oversampled test corpus *C*_test-i_ and *L*_test-i_ were used to test the trained models.

## Results

**Figure 1:**
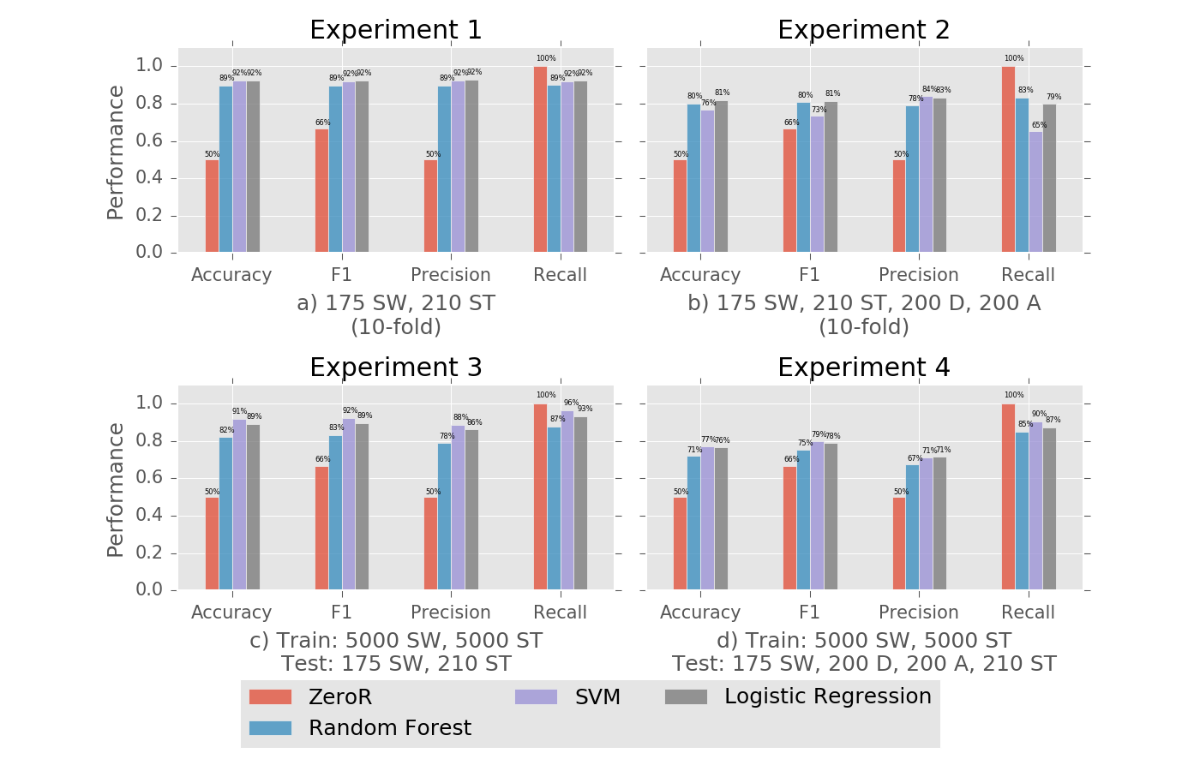
Prediction performance evaluation for the four experiments with different combinations of posts from SuicideWatch (SW), Depression (D), Anxiety (A), ShowerThoughts (ST) subreddits.

In all the experiments, logistic regression and SVM (except for experiment 2) performed the best, followed by random forest, all much more performant than ZeroR, which provided a baseline (66% for F_1_, 50% for other metrics) for performance evaluation of the classification task (see Figure 1). Although SVM performance slightly exceeded logistic regression in experiments 3 and 4, logistic regression would be favorable due to its simplicity.

In experiment 1, the logistic regression and SVM classifiers could differentiate suicidal posts from nonsuicidal posts with an F_1_ score of 92%, followed by random forest (89%). We can attribute this performance to the suicidality levels of SuicideWatch and ShowerThoughts being on different sides of the scale. The experiment yielded good results as expected. Furthermore, if posts in SuicideWatch mentioning suicidal people other than the author were annotated as suicidal, those scores would be even higher. When LIWC and sentiment features were removed from the feature set, F_1_ score went down from 92% to 88%, which is not a significant decrease. This shows that results are still promising even when solely tf-idf matrices were used.

When the Depression and Anxiety subreddits were introduced in the experiment 2 dataset, the performance was 11% lower for logistic regression (F_1_/accuracy=81%), followed by random forest (F_1_/accuracy=80%) and SVM (F_1_=73%, accuracy=76%), but still sufficiently high when compared to the baseline classifier (F_1_=66%, accuracy=50%). This also complies with our expectations because the algorithm had to deal with edge cases where depressive and suicidal people have a large intersection in vocabulary. With more labeled data, a better generalization can be achieved.

In experiment 3, where the models were trained with nonannotated posts and tested against annotated posts, F_1_ and accuracy scores were 89% for logistic regression, 3% less than experiment 1. This was an expected reduction because some of the posts in SuicideWatch had suicidal context but were not words of a suicidal person. Nevertheless, lack of annotated posts in the training set was compensated with a higher number of posts from both subreddits, yielding a better generalization. On the other hand, SVM could surpass logistic regression with F_1_=92%.

In experiment 4, lack of posts for training edge cases (annotated posts in Depression and Anxiety) caused a lower but still acceptable prediction performance (an F_1_ score of 78% against 66% and an accuracy of 77% against 50%) than in experiment 3. This was an expected result and can be improved by feeding annotated posts from Depression and Anxiety into the training set.

## Discussion

### Principal Results

The high (100%) recall performance of the baseline classifier ZeroR is due to its strategy to predict posts as suicidal all the time. This strategy comes with a penalty of false positive overhead, thus 0% precision. This means providing psychological support to everyone, regardless of the content of their posts, which is practically useless, potentially harmful, and costly. The high recall rate—which should be ignored during evaluation for ZeroR—supports a higher than expected illusional F_1_ score (66%) in all cases. Although F_1_ score is the widely used metric due to its balancing nature, other parameters including accuracy (which is valuable when the dataset is balanced) and precision (to observe the overhead) should be used for comparison with the baseline classifier.

In all the experiments, logistic regression and SVM performed much better than ZeroR baseline algorithm. In the first experiment, the classifiers could predict suicidality with an F_1_ score of 92%. There was a reduction in performance when annotated Depression and Anxiety subreddit posts were introduced in experiment 2. This is due to posts in these two subreddits having gradient levels of suicidality, introducing new edge cases the algorithm should handle, making it harder to differentiate nonsuicidal depressives from suicidal people. Solely looking at the vocabulary and psychological meanings of the words seems to have confused the classifier, suggesting a more contextual approach, more data, and maybe a deeper classifier might be required to obtain performance levels in the first experiment.

The high performance in the third experiment shows the assumptions of (1) posts in SuicideWatch subreddit being suicidal and (2) posts in ShowerThoughts being nonsuicidal was valid. It can be said that these assumptions hold when discriminating suicidal posts from nonsuicidal posts in the absence of edge cases such as in Depression and Anxiety posts. When the aforementioned edge cases are introduced in the test set but not in the training set of experiment 4, the performance was lower (which was expected), although still significantly higher than ZeroR algorithm.

### Practical Use

Our findings show that text mining methods can be used to detect posts with suicidal ideation online. Being one of the simplest, efficient, and most interpretable models, logistic regression performs very well on the problem. The model trained in this study can be used in spotting people with suicidal ideation while they are writing their forum (or blog) posts right away using a Javascript or mobile app library. Popup dialogs can be shown to authors of posts classified as suicidal. Authors can be asked how they feel and whether they need help or have suicidal thoughts without irritating or leading them to nonexistent suicidal thoughts. Thanks to interpretability and simplicity of logistic regression, the code to embed on mobile apps, blogs, forums, or even Web browsers would add very little overhead. On admission of having suicidal thoughts on the popup, the author can be offered support immediately via live chat, phone call, or face-to-face counseling. Accepting the offer would be a verification of our prediction and false positives would come mainly from depressed people who have premature suicidal thoughts (who should be supported as well); therefore, lowering precision levels would not pose an overhead on the support staff. This means the model can be tuned further in favor of recall, instead of precision, by changing threshold values of logistic regression. Experiment 3 has validated the assumption that posts on SuicideWatch subreddit can be assumed as suicidal. Thus, a logistic regression model trained as in experiment 3 with the whole SuicideWatch and ShowerThoughts data would be sufficient for a real-world application since the verification system would eliminate the false positives. With the introduction of annotated edge cases (from Depression and Anxiety subreddits) to the training set, the performance can be further improved. Another interesting strategy would be using the responses received from the authors (subject to this prevention system) as annotations to further train the model to make better predictions, leading to an ever-learning online classifier.

### Limitations

The prediction system in study is limited to text posts in the English language. Similar models can be trained on other languages given sufficient dataset. Without knowledge of whether the authors of the posts committed suicide, our system can only claim to predict suicidal ideation, not a potential suicide attempt. Although our dataset is limited to Reddit, which is a forum itself, we expect our system to work well on other forums and blogs due to similarity in format and context. However, further research is needed to verify this claim. This study is a proof of concept for online suicidal ideation surveillance, yet further development is needed for a real-time online suicide prevention system after designing appropriate questionnaires to be asked to authors with suicidal markers. In all, 175 SuicideWatch posts were annotated by psychiatrists. Due to the time-consuming nature of annotating hundreds of posts, the rest of the subreddits were annotated by AEA under guidance of the psychiatrists NBA and OZ. To avoid introducing a bias, AEA performed annotation on SuicideWatch posts as well and the similarity between annotations of authors were analyzed. It was seen that AEA and the psychiatrists agreed on annotations 87% and 89% of the time, whereas the psychiatrists agreed 93% of the time among themselves before resolving conflicts. So this indicates annotations of the computer scientist (AEA) are not expected to introduce bias.

### Conclusions

To the best of our knowledge, this is the first study that uses forum posts in thousands scale (of which 785 were manually annotated) with an objective of detecting posts with suicidal ideation with high performance in all metrics. Results indicate it is possible to detect suicidal people online to provide them proper immediate support as they are writing. Authors of this text acknowledge that detecting suicidal ideation with high accuracy is a difficult problem even for humans and design of nonintrusive conversation for potential suicidal candidates should be carried out carefully. Application of such a detection system in real time may save thousands of lives every year if carried out properly.

## Appendix

Multimedia Appendix 1 Method Descriptions.

Multimedia Appendix 2 Feature Extraction and Experiment Design Diagram.

## Abbreviations

LIWC: linguistic inquiry and word count
NLTK: Natural Language Toolkit
SVM: support vector machine
tf-idf: term frequency-inverse term frequency
