# Supplementary material for: Detecting Suicidal Ideation on Forums: Proof-of-Concept Study
Source: J Med Internet Res. 2018 Jun 21;20(6):e215. doi: 10.2196/jmir.9840 (PMC6035349; doi:10.2196/jmir.9840)
Supplement: Multimedia Appendix 1 [file jmir_v20i6e215_app1.pdf]

Multimedia Appendix 1

Introduction

Text Mining Methods for Suicidal Ideation Detection

Term Frequency (Bag of Words) Approach

Figure 1: Sample Document Term Matrix with Bag of Words approach with hypothetical documents and terms.

	t_1	t_2	t_3	...	t_N	total
d_1	3 / 130	4 / 130	2 / 130	...	6 / 130	130
d_2	10 / 1510	13 / 1510	47 / 1510	...	10 / 1510	1510
...	...	...	...	...	...	...
d_M	tf-idf(t_1, d_M)	tf-idf(t_2, d_M)	tf-idf(t_3, d_M)	...	tf-idf(t_N, d_M)	...

Bag of words method [17] builds a matrix called *Document Term Matrix* (see Figure 1) where rows correspond to documents, columns correspond to terms (usually words in stemmed format) and cells correspond to term frequency of the word in that document. Having several variations, term frequency $tf(t, d)$ in the simplest definition, is the number of occurrences of a

term t in document d divided by the number of all terms in the document. Suppose $D = \{d_1, d_2, \ldots, d_M\}$is a set of documents. Let $T = \{t_1, t_2, \ldots, t_N\}$ be the set of terms in these documents. The number of occurrences of t_i in d_j is calculated and normalized by dividing the number of occurrences of all terms in d_j. This normalized ratio is called term frequency, denoted by $tf(t_i, d_j)$. The ratio is high if the term is frequently occurs in the document, which indicates that the document is related to that term. If there is no other term in the document, then the ratio becomes 1, which is the maximum possible value. For instance, in the hypothetical text represented with Figure 1, term t_1 occurs 3 times in document d_1 which contains a total of 130 terms, resulting in a term frequency of 3 / 130 = 0.023. By using bag of words approach, frequently used terms in suicidal or non-suicidal posts can be found and used to discriminate these two classes. However some terms like "the" are used very frequently in all kinds of documents, putting on noise to the statistics, reducing the importance of other terms. To avoid this, inverse document frequency (idf), which reduces the importance of commonly used terms, is commonly incorporated in addition to term frequency.

Tf-Idf Approach

In tf-idf approach [18], score for a cell is calculated by multiplying term frequency tf for the [term t, document d] pair with *inverse document frequency (idf)* scores for the term t over document space D: $tf - idf(t, d, D) = tf(t, d) * idf(t, D)$. Inverse document frequency represents how less-frequently a term is used across all the documents (D). It is defined as the logarithmic inverse frequency of a given term t among documents $d \in D$. This score can be calculated by $idf(t, D) = log \frac{|D|}{1 + |\{d \in D : t \in D\}|}$. It allows decreasing importance of

words that are commonly used in several posts and hence that don't have much importance (like commonly used stop words) when multiplied with term frequency.

LIWC Approach

One of the promising tools, LIWC is a commonly used text analysis program that counts words in psychologically meaningful categories and yielding a score for each category. It builds a matrix where rows correspond to documents, columns correspond to categories and cells correspond to score calculated for that document-category pair. In this sense, LIWC is an important tool in capturing correlations between choices of words and suicidality. It provides scores in 93 categories and Pennebaker [19] listed them as:

> "Word count, 4 summary language variables (analytical thinking, clout, authenticity, and emotional tone), 3 general descriptor categories (words per sentence, percent of target words captured by the dictionary, and percent of words in the text that are longer than six letters), 21 standard linguistic dimensions (e.g., percentage of words in the text that are pronouns, articles, auxiliary verbs, etc.), 41 word categories tapping psychological constructs (e.g., affect, cognition, biological processes, drives), 6 personal concern categories (e.g., work, home, leisure activities), 5 informal language markers (assents, fillers, swear words, netspeak), and 12 punctuation categories (periods, commas, etc)."

With these statistics, it is possible to extract psychological mood or the main concerns of a post author which helps predicting suicidality. One pitfall is that LIWC might not be ideal for real time predictions due to non-automated nature of LIWC. It requires a human to run a desktop

application to calculate scores. This requirement makes LIWC tool hard to run it for different inputs in an automated fashion.

Sentiment Analysis Approach

Similarly, Sentiment Analysis is the process of determining the emotional tone behind a series of words using Natural Language Processing (NLP) techniques. As a result of this process, polarity and subjectivity scores are produced for each document, resulting in a 2-column matrix. *Polarity score* is a real number between -1 (negative) and 1 (positive), representing the degree the document is positive. *Subjectivity score*, on the other hand, is a real number between 0 (factual) and 1 (totally subjective). Selected feature columns and values resulting from these processes are combined and used for prediction modeling. Sentiment features allow detecting negative mood which is significantly common among people with suicidal ideation.

Methods

Data Collection

Reddit.com, founded in 2005, is one of the most popular forum sites as of 2018. It is comprised of several subreddits (sections) on specific topics and themes on which millions of people have conversations. Some examples to subreddits are: Science, Technology, Sports, Romance, Funny, Worldnews, Books, Fitness, Documentaries, ShowerThoughts, Anxiety, Depression, SuicideWatch. As of 2017, Reddit had 250 million users from more than 209 countries and more than 190 million posts (majority

in English language), making it a very important data source for researchers who want to apply text mining to extract opinions.

Results

In addition to accuracy, recall and precision, below are the false positive and false negative prediction performances. False positive indicates the ratio of posts that were actually non-suicidal but the algorithm classified as suicidal. False negative indicates the ratio of posts that were actually suicidal but the algorithms missed (classified as non-suicidal). In suicide prevention scope, keeping false negatives low is more important than keeping false positives low. However a 100% false positive would mean allocating resources for all the posts.

Figure 2: False positive rates (FPR) and false negative rates (FNR) for classifications. It can be seen that LR and SVM are performing the best (except for Experiment 2 where SVM keeps behind of LR and RF in terms of FNR).

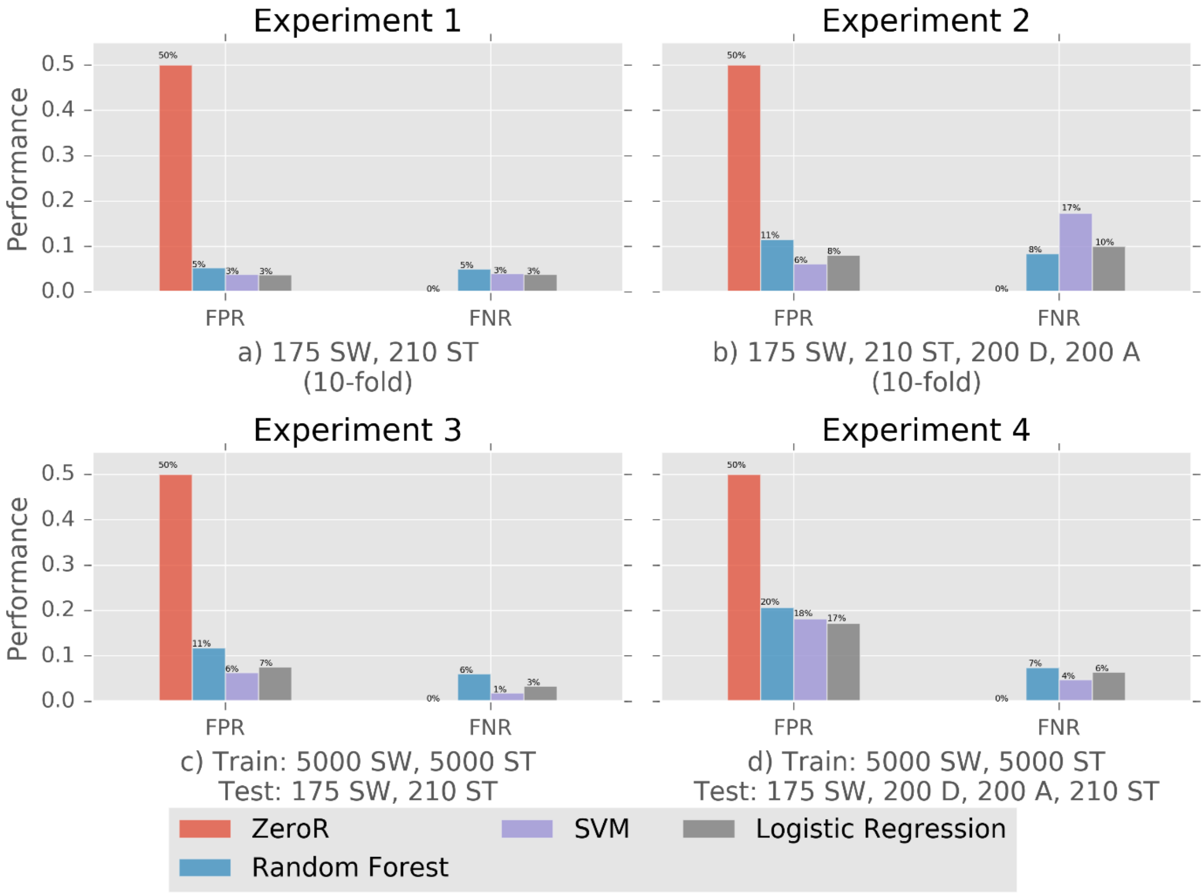

Experiment 1
Experiment 2
Experiment 3
Experiment 4
Performance
0.0
0.1
0.2
0.3
0.4
0.5
FPR
FNR
a) 175 SW, 210 ST
(10-fold)
b) 175 SW, 210 ST, 200 D, 200 A
(10-fold)
c) Train: 5000 SW, 5000 ST
Test: 175 SW, 210 ST
d) Train: 5000 SW, 5000 ST
Test: 175 SW, 200 D, 200 A, 210 ST
ZeroR
Random Forest
SVM
Logistic Regression
